# Testing moderator hypotheses in meta-analytic structural equation modeling using subgroup analysis

**DOI:** 10.3758/s13428-018-1046-3

**Published:** 2018-06-04

**Authors:** Suzanne Jak, Mike W.-L. Cheung

**Affiliations:** 1 Methods and Statistics, Child Development and Education, University of Amsterdam, Nieuwe Achtergracht 127, 1018 WS Amsterdam, The Netherlands; 2 National University of Singapore, Singapore, Singapore

**Keywords:** Meta-analytic structural equation modeling, Two-stage structural equation modeling, Meta-analysis, Random-effects model, Subgroup analysis

## Abstract

Meta-analytic structural equation modeling (MASEM) is a statistical technique to pool correlation matrices and test structural equation models on the pooled correlation matrix. In Stage 1 of MASEM, correlation matrices from independent studies are combined to obtain a pooled correlation matrix, using fixed- or random-effects analysis. In Stage 2, a structural model is fitted to the pooled correlation matrix. Researchers applying MASEM may have hypotheses about how certain model parameters will differ across subgroups of studies. These moderator hypotheses are often addressed using suboptimal methods. The aim of the current article is to provide guidance and examples on how to test hypotheses about group differences in specific model parameters in MASEM. We illustrate the procedure using both fixed- and random-effects subgroup analysis with two real datasets. In addition, we present a small simulation study to evaluate the effect of the number of studies per subgroup on convergence problems. All data and the R-scripts for the examples are provided online.

The combination of meta-analysis and structural equation modeling (SEM) for the purpose of testing hypothesized models is called meta-analytic structural equation modeling (MASEM). Using MASEM, correlation matrices from independent studies can be used to test a hypothesized model that explains the relationships between a set of variables or to compare several alternative models that may be supported by different studies or theories (Viswesvaran & Ones, 1995). The state-of-the-art approach to conducting MASEM is the two-stage SEM (TSSEM) approach (Cheung, 2014; Cheung & Chan, 2005b). In the first stage of the analysis, correlation matrices are combined to form a pooled correlation matrix with a random- or fixed-effects model. In the second stage of the analysis, a structural equation model is fitted to this pooled correlation matrix. Several alternative models may be tested and compared in this stage. If all variables were measured on a common scale across studies, analysis of covariance matrices would also be possible (Cheung & Chan, 2009). This would allow researchers to study measurement invariance across studies. In this paper we focus on correlation matrices although the techniques that are discussed are directly applicable to covariance matrices.

Researchers often have hypotheses about how certain parameters might differ across subgroups of studies (e.g., Rosenbusch, Rauch, and Bausch (2013)). However, there are currently no straightforward procedures to test these hypotheses in MASEM. The aims of the current article are therefore: 1) to provide guidance and examples on how to test hypotheses about group differences in specific model parameters in MASEM; 2) to discuss issues with regard to testing differences between subgroups based on pooled correlation matrices; and 3) to show how the subgroup models with equality constraints on some parameters can be fitted using the metaSEM (Cheung, 2015b) and OpenMx packages (Boker et al., 2014) in R (R Core Team, 2017).

Specifically, we propose a follow-up analysis in which the equality of structural parameters across studies can be tested. Assuming that there are hypotheses on categorical study-level variables, the equality of specific parameters can be tested across subgroups of studies. In this way, it is possible to find a model in which some parameters are equal across subgroups of studies and others are not. More importantly, it helps researchers to identify how study-level characteristics can be used to explain differences in parameter estimates.

## Methods to model heterogeneity in meta-analysis

With regard to how to handle heterogeneity in a meta-analysis, two dimensions (or approaches) can be distinguished (e.g., Borenstein, Hedges, Higgins, and Rothstein (2009)). The first dimension concerns whether to apply a fixed- or a random-effects model, while the second dimension is about whether or not to include study-level moderators. Two classes of models can be differentiated: the fixed-effects model and the random-effects model. The fixed-effects model allows conditional inference, meaning that the results are only relevant to the studies included in the meta-analysis. The random-effects model allows for unconditional inference to studies that could have been included in the meta-analysis by assuming that the included studies are samples of a larger population of studies (Hedges & Vevea, 1998).

The fixed-effects model (without moderators) usually assumes that all studies share the same population effect size, while the fixed-effects model with moderators assumes that the effects are homogeneous after taking into account the influence of moderators. The random-effects model assumes that the differences across studies are random. The random-effects model with moderators, known as a mixed-effects model, assumes that there will still be random effects after the moderators are taken into account.

### Methods to model heterogeneity in MASEM

The above framework from general meta-analysis is also applicable to MASEM. Table 1 gives an overview of the suitability, and the advantages and disadvantages of using different combinations of fixed- versus random-effects MASEM, with or without subgroups. Case 1 represents overall analysis with a fixed-effects model. Fixed-effects models are very restrictive, (i.e. the number of parameters to be estimated is relatively small), which makes them easy to apply. However, homogeneity of correlation matrices across studies may not be realistic, leading to biased significance tests (Hafdahl, 2008; Zhang, 2011).

**Table 1:** Overview of advantages (+) and disadvantages (–) of subgroup versus overall analysis and fixed-effects versus random-effects models

		FEM	REM
		Case 1	Case 2
	Use if:	There is no hypothesis about moderation, and homogeneity is realistic	There is no hypothesis about moderation, and homogeneity is not realistic
Overall	+	1) Small number of parameters	1) Accounts for heterogeneity
		2) Sometimes the only option (e.g. with a small number of studies)	2) Allows for unconditional inference
	–	1) Only allows for conditional inference	1) Large number of parameters (but smaller than without subgroups)
		2) Biased significance tests if homogeneity does not hold	2) No information about specific effects of moderators
		3) Masks subgroup differences in parameters	3) Masks subgroup differences in parameters
		Case 3	Case 4
	Use if:	There is a specific hypothesis about subgroups, and homogeneity within subgroups is realistic	There is a specific hypothesis about subgroups, and homogeneity within subgroups is not realistic
Subgroups	+	1) Small number of parameters	1) Accounts for additional heterogeneity within subgroups
		2) Sometimes the only option (e.g. with a small number of studies)	2) Allows for unconditional inference
		3) Posibility to test subgroup differences in parameters	3) Posibility to test subgroup differences in parameters
	–	1) Only allows for conditional inference	1) Large number of parameters (larger than without subgroups)
		2) Need to dichotomize continuous moderator	2) Need to dichotomize continuous moderator
		2) Biased parameter estimates if homogeneity does not hold	3) Number of studies per subgroup might get too small

One way to account for heterogeneity is by estimating between-study heterogeneity across all studies in the random-effects approach (Case 2 in Table 1). By using a random-effects model, the between-study heterogeneity is accounted for at Stage 1 of the analysis (pooling correlations), and the Stage 2 model (the actual structural model of interest) is fitted on the averaged correlation matrix. Under the random-effects model, study-level variability is considered a nuisance. An overall random-effects analysis may be the preferred choice when moderation of the effects by study-level characteristics is not of substantive interest (Cheung & Cheung, 2016).

Subgroup analysis is more appropriate than overall random-effects analysis in cases where it is of interest to determine how the structural models differ across levels of a categorical study-level variable, (Cases 3 and 4 in Table 1). In a subgroup analysis, the structural model is fitted separately to groups of studies. Within the subgroups, one may use random- or fixed-effects modeling (Jak, 2015). Fixed-effects subgroup analysis is suitable if homogeneity of correlations within the subgroups is realistic. Most often, however, heterogeneity within subgroups of studies is still expected, and fixed-effects modeling may be unrealistic. In such cases, random-effects subgroup analysis may be the best choice. A possible problem with a random-effects subgroup analysis is that the number of studies within each subgroup may become too small for reliable results to be obtained.

We focus on the situation in which researchers have an *a priori* idea of which study-level characteristics may moderate effects in the Stage 2 model. That is, we do not consider exploratory approaches, such as using cluster analysis to find homogeneous subgroups of studies (Cheung & Chan, 2005a).

Besides the random-effects model and subgroup analysis, Cheung and Cheung (2016) discuss an alternative approach to addressing heterogeneity in MASEM, called “parameter-based MASEM”. Since this approach also has its limitations, and discussing them is beyond the scope of the current work, we refer readers to their study for more details. We focus on TSSEM, in which subgroup analysis is the only option to evaluate moderator effects.

### Currently used methods to test hypotheses about heterogeneity in MASEM

A disadvantage of the way subgroup analysis is commonly applied, is that all Stage 2 parameters are allowed to be different across subgroups, regardless of expectations about differences in specific parameters. That is, differences in parameter estimates across groups are seldom tested in the structural model. For example, Rosenbusch et al., (2013) performed a MASEM analysis on data from 83 studies, testing a model in which the influence of the external environment of firms on performance levels is mediated by the entrepreneurial orientation of the firm. They split the data into a group of studies based on small sized firms and medium-to-large sized firms, to investigate whether the regression parameters in the path model are moderated by firm size. However, after fitting the path model to the pooled correlation matrices in the two subgroups, they compared the results without using any statistical tests.

Gerow et al., (2013) hypothesized that the influence of intrinsic motivation on individuals’ interaction with information technology was greater when the technology was to be used for hedonistic applications than for practical applications. They fitted the structural model to a subgroup of studies with hedonistic applications, a subgroup of studies with practical applications, and a subgroup of studies with a mix of applications. However, to test for differences between the subgroups, they performed t-tests on the four pooled Stage 1 correlation coefficients in the subgroups, ignoring the estimates in the actual path models altogether. These approaches are not ideal because researchers cannot test whether some of the parameters, those that may be of theoretical interest, are significantly different across groups.

More often than using subgroup analysis, researchers address the moderation of effect sizes using standard meta-analysis techniques on individual effect sizes, before they conduct the MASEM analysis. They use techniques such as meta-regression or ANOVA-type analyses (Lipsey and Wilson, 2001). Independent of the moderation effects, the MASEM is then performed using the full set of studies. Examples of this practice can be found in Drees and Heugens (2013), Earnest, Allen, and Landis (2011) and Jiang, Liu, Mckay, Lee, and Mitchell (2012). A disadvantage of this approach is that moderation is tested on the correlation coefficients, and not on specific parameters in a structural equation model. Most often, this is not in line with the hypothesis of interest. For example, the moderator hypotheses of Gerow et al., (2013), were about the direct effects in the path model but not about covariances and variances. Although subgroup analysis to test heterogeneity has previously been conducted (see Haus et al. 2013), we think that instructions regarding the procedures are needed because most researchers who apply MASEM still choose to address issues of moderation outside the context of MASEM.

## Overview of this article

In the next sections, we briefly introduce fixed- and random-effects TSSEM and propose a follow-up analysis to address heterogeneity using subgroup analysis. We discuss some issues related to testing the equality of parameters using pooled correlation matrices. Next, we illustrate the procedure using an example of testing the equality of factor loadings across study-level variables of the Hospital Anxiety and Depression Scale (HADS) with data from Norton, Cosco, Doyle, Done, and Sacker (2013) as well as with an example of testing moderation by socio-economic status (SES) in a path model linking teacher-child relations to engagement and achievement (Roorda, Koomen, Spilt, & Oort, 2011). To facilitate the use of the proposed procedure, detailed reports of the analyses, including data and R-scripts, are provided online at www.suzannejak.nl/masem_code. Finally, we present a small simulation study to evaluate the effect of the number of studies included in a MASEM analysis on the frequency of estimation problems.

## TSSEM

In the next two sections we briefly describe fixed-effects TSSEM and random-effects TSSEM. For a more elaborate explanation see Cheung and Chan (2005b), Cheung (2014), Cheung (2015a), and Jak (2015).

### Fixed-effects TSSEM

The fixed-effects TSSEM approach was proposed by Cheung and Chan (2005b). They performed a simulation study, comparing the fixed-effects TSSEM approach to two univariate approaches (Hunter & Schmidt, 2015; Hedges & Olkin, 1985) and the multivariate GLS-approach (Becker, 1992, 1995). They found that the TSSEM approach showed the best results with respect to parameter accuracy and false positive rates of rejecting homogeneity.

#### Stage 1

In fixed-effects TSSEM, the correlation matrices in the individual studies are assumed to be homogenous across studies, all being estimates of one common population correlation matrix. Differences between the correlation matrices in different studies are assumed to be solely the result of sampling error. The model that is fitted at Stage 1 is a multigroup model in which all correlation coefficients are assumed to be equal across studies. Fitting this model to the observed correlation matrices in the studies leads to an estimate of the population correlation matrix $\mathbf {P}_{\mathrm {F}}$, which is correctly estimated if homogeneity indeed holds.

#### Stage 2

In Stage 2 of the analysis, weighted least squares (WLS) estimation (Browne, 1984) is used to fit a structural equation model to the estimated common correlation matrix from Stage 1. The proposed weight matrix in WLS-estimation is the inverse asymptotic variance covariance matrix of the Stage 1 estimates of $\mathbf {P}_{\mathrm {F}}$, i.e., $\mathbf {W}_{\mathrm {F}}=\mathbf {V}^{-1}_{\mathrm {F}}$ (Cheung & Chan, 2005b). These weights ensure that correlation coefficients that are based on more information (on more studies and/or studies with larger sample sizes) get more weight in the estimation of the Stage 2 parameters. The Stage 2 analysis leads to estimates of the model parameters and a $\chi ^{2}$ measure of fit.

### Random-effects TSSEM

#### Stage 1

In random-effects TSSEM, the population effects sizes are allowed to differ across studies. The between-study variability is taken into account in the Stage 1 analysis. Estimates of the means and the covariance matrices in random-effects TSSEM are obtained by fixing the sampling covariance matrices to the known values (through definition variables, see Cheung (2015a), and using full information maximum likelihood to estimate the vector of means, $\mathbf {P}_{\mathrm {R}}$, and the between-studies covariances, $\mathbf {T}^{2}$ (Cheung, 2014).

#### Stage 2

Fitting the Stage 2 model in the random-effects approach is not very different from fitting the Stage 2 model in the fixed-effects approach. The values in $\mathbf {W}_{\mathrm {R}}$ from a random-effects analysis are usually larger than those obtained from a fixed-effects analysis, because the between-studies covariance is added to the construction of the weight matrix. This results in relatively more weight being given to smaller studies, and larger standard errors and confidence intervals, than with the fixed-effects approach.

## Using subgroup analysis to test parameter heterogeneity

The basic procedure for subgroup analysis comprises separate Stage 1 analyses for the subgroups. The Stage 1 analyses may be in the fixed-effects framework, hypothesizing homogeneity within subgroups, or in the random-effects framework, assuming that there is still substantive between-study heterogeneity within the subgroups. In a subgroup MASEM analysis, it is straightforward to equate certain parameters across groups at Stage 1 or Stage 2 of the analysis. The differences in the parameters across groups can be tested using a likelihood ratio test by comparing the fit of a model with across-groups equality constraints on certain parameters with a model in which the parameters are freely estimated across groups.

### Testing heterogeneity in Stage 1 parameters

Although we focus on testing differences in Stage 2 parameters, in some situations it may be interesting to test the equality of the pooled correlation matrices across subgroups. In order to test the hypothesis that the correlation matrices from a fixed-effects subgroup analysis, $\mathbf {P}_{\mathrm {F}}$, are equal across subgroups *g*, one could fit a model with the constraint $\mathbf {P}_{\mathrm {F_{g1}}}$ = $\mathbf {P}_{\mathrm {F_{g2}}}$. Under the null hypothesis of equal correlation matrices across groups, the difference in the -2 log-likelihoods of the models with and without this constraint asymptotically follows a chi-square distribution with degrees of freedom equal to the number of constrained correlation coefficients. Similarly, one could perform this test on the averaged correlation matrices from a random-effects Stage 1 analysis. With random-effects analysis, it may additionally be tested if the subgroups differ in their heterogeneity covariance matrices $\mathbf {T}^{2}_{g}$. When the researcher’s hypotheses are directly about Stage 2 parameters, one may skip testing the equality of equal correlation matrices across subgroups. The equality of between-studies covariance matrices may still be useful to reduce the number of parameters to be estimated in a random-effects analysis. This issue is discussed further in the general discussion.

### Testing heterogeneity in Stage 2 parameters

For ease of discussion, we suppose that there are two subgroups. Given the two Stage 1 pooled correlation matrices in the subgroups *g*, say, $\mathbf {P}_{g}$, a structural model can be fitted to the two matrices. For example, one could fit a factor model in both groups:

1 $$ \mathbf{P}_{g} = \boldsymbol{\Lambda}_{g}\, \boldsymbol{\Phi}_{g}\, \boldsymbol{\Lambda}_{g}^{T}\, + \boldsymbol{\Psi}_{g}\, , $$

where with *p* observed variables and *k* common factors, $\boldsymbol {\Lambda }_{g}$ is a full *p* by *k* matrix with factor loadings in group *g*, $\boldsymbol {\Phi }_{g}$ is a *k* by *k* symmetrical matrix with factor variances and covariances in group *g*, and $\boldsymbol {\Psi }_{g}$ is a *p* by *p* symmetrical matrix with residual (co)variances in group *g*. The covariance structure is identified by setting diag(**Φ**_*g*_) = **I**. Since the input is a correlation matrix, the constraint diag(**P**_*g*_) = **I**, is required to ensure that the diagonals of $\mathbf {P}_{g}$ are always ones during estimation.

In order to test the equality of factor loadings across groups, a model can be fitted in which $\boldsymbol {\Lambda }_{g}$ = $\boldsymbol {\Lambda }$. Under the null hypothesis of equal factor loadings, the difference in chi-squares of the models with $\boldsymbol {\Lambda }_{g}$ = $\boldsymbol {\Lambda }_{g}$ and $\boldsymbol {\Lambda }_{g}$ = $\boldsymbol {\Lambda }$ asymptotically follows a chi-square distribution with degrees of freedom equal to the difference in the number of freely estimated parameters. If the difference in chi-squares is considered significant, the null hypothesis of equal factor loadings is rejected.

The approach of creating subgroups with similar study characteristics and equating parameters across groups is suitable for any structural equation model. For example, in a path model, it may be hypothesized that some or all direct effects are different across subgroups of studies, but variances and residual variances are not. One could then compare a model with equal regression coefficients with a model with freely estimated regression coefficients to test the hypothesis. Also, the subgroups approach can be applied using fixed-effects or random-effects analyses.

### Issues related to testing equality constraints based on correlation matrices in TSSEM

Structural equation models are ideally fitted on covariance matrices. In MASEM, and meta-analysis in general, it is very common to synthesize correlation coefficients. One reason for the synthesis of standardized effect sizes is that different studies may use different instruments with different scales to operationalize the variables of interest. The analysis of correlation matrices does not pose problems when the necessary constraints are included (Bentler and Savalei, 2010; Cheung, 2015a). However, it should be taken into account that fitting models to correlation matrices with TSSEM implies that all parameter estimates are in a standardized metric (assuming that all latent variables are scaled to have unit variances, which is recommended in TSSEM (Cheung, 2015a)).

When we compare models across subgroups in TSSEM, we are thus comparing parameter estimates that are standardized with respect to the observed and latent variables within the subgroups (Cheung, 2015a; Steiger, 2002). This may not necessarily be a problem - sometimes it is even desirable to compare standardized coefficients (see Kwan and Chan (2011)). For example, van den Boer, van Bergen, and de Jong (2014) tested the equality of correlations between three reading tasks across an oral and a silent reading group. However, it is important to be aware of this issue and to interpret the results correctly. Suppose that a standardized regression coefficient from variable *x* on variable *y* $\beta ^{*}_{yx}$, is compared across two subgroups of studies, $g_{1}$ and $g_{2}$. The standardized direct effects in the subgroups are given by:

2 $$ \beta^{*}_{yx_{g1}} = \beta_{yx_{g1}} \frac{\sigma_{x_{g1}}}{\sigma_{y_{g1}}} $$

and

3 $$ \beta^{*}_{yx_{g2}} = \beta_{yx_{g2}} \frac{\sigma_{x_{g2}}}{\sigma_{y_{g2}}} , $$

where $\beta $ represents an unstandardized regression coefficient, $\beta ^{*}$ represents a standardized regression coefficient, and $\sigma $ represents a standard deviation. In the special case that the standard deviations of *x* and *y* are equal within subgroups, in each subgroup the standardized coefficient is equal to the unstandardized coefficient, and the test of $H_{0}$: $\beta ^{*}_{yx_{g1}}$ = $\beta ^{*}_{yx_{g2}}$ is equal to the test of $H_{0}$: $\beta _{yx_{g1}}$ = $\beta _{yx_{g2}}$. In fact, this not only holds when the standard deviations of the variables are equal in the subgroups, but in general when the ratio of $\sigma _{x}$ over $\sigma _{y}$ is equal across subgroups. For example, when $\sigma _{x}$ and $\sigma _{y}$ in group 1 are respectively 2 and 4, and the $\sigma _{x}$ and $\sigma _{y}$ in group 2 are respectively 1 and 2, the standardized regression coefficient equals the unstandardized coefficient times .5 in both groups. In this case, a test of the equality of the standardized regression coefficients will lead to the same conclusion as a test of the unstandardized regression coefficients.

However, in most cases the ratio of standard deviations will not be exactly equal across groups. Therefore, when testing the equality of regression coefficients in a path model, one has to realize that all parameters are in a standardized metric. The conclusions may not be generalizable to unstandardized coefficients. Whether the standardized or the unstandardized regression coefficients are more relevant depends on the research questions (Bentler, 2007). In the context of meta-analysis, standardized coefficients are generally preferred (Cheung, 2009; Hunter & Hamilton, 2002).

In a factor analytic model, several methods of standardization exist. Parameter estimates may be standardized with respect to the observed variables only, or with respect to the observed variables and common factors. In MASEM, it is recommended that the common factors be identified by fixing their variances to 1 (Cheung, 2015a). All results obtained from a MASEM-analysis on correlation matrices are thus standardized with respect to the observed variables and the common factor. As a consequence of this standardization, the residual variances in $\boldsymbol {\Psi }$ are effectively not free parameters, but the remainder of $diag(\mathbf {I}) - diag(\boldsymbol {\Lambda } \boldsymbol {\Phi } \boldsymbol {\Lambda }^{T}$) (Cheung, 2015a).

Similar to path analysis, when testing the equality of factor loadings across subgroups in MASEM, the results may not be generalizable to unstandardized factor loadings, due to across-group differences in the (unknown) variances of the indicators and common factors. Moreover, if all standardized factor loadings are set to be equal across groups, this implies that all standardized residual variances are equal across groups. Note that although one may be inclined to denote a test of the equality of factor loadings a test of weak factorial invariance (Meredith, 1993), this would strictly be incorrect, as weak factorial invariance pertains to the equality of unstandardized factor loadings.

## Examples

In this section, we present two examples of the testing of moderator hypotheses in MASEM using subgroup analysis. Example 1 illustrates the testing of the equality of factor loadings using factor analysis under the fixed-effects model (Case 1 and 3 from Table 1). Example 2 illustrates the testing of the moderation of direct effects using path analysis under the random-effects model (Case 2 and 4 from Table 1). The R-syntax for the examples can be found online (http://www.suzannejak.nl/masem_code).

### Example 1 – Testing equality of factor loadings of the Hospital Anxiety and Depression Scale

#### Introduction

The HADS was designed to measure psychological distress in non-psychiatric patient populations (Zigmond & Snaith, 1983), and is widely used in research on distress in patients. The instrument consists of 14 items: the odd numbered items are designed to measure anxiety and the even numbered items are designed to measure depression. The items are scored on a 4-point scale. Some controversy exists regarding the validity of the HADS (Zakrzewska, 2012). The HADS has generally been found to be a useful instrument for screening purposes, but not for diagnostics purposes (Mitchell, Meader, & Symonds, 2010). Ambiguous results regarding the factor structure of the HADS led to a meta-analytic study by Norton et al., (2013), who gathered correlation matrices of the 14 HADS items from 28 published studies. Using meta-analytic confirmatory factor analysis, they found that a bi-factor model that included all items loading onto a general distress factor and two orthogonal anxiety and depression factors provided the best fit to the pooled data. Of the 28 studies evaluated by Norton et al., 10 considered non-patient samples and 18 were based on patient samples. As an illustration we will test the equality of factor loadings across studies based on patient and non-patient samples.

#### Analysis

**Fig. 1:**
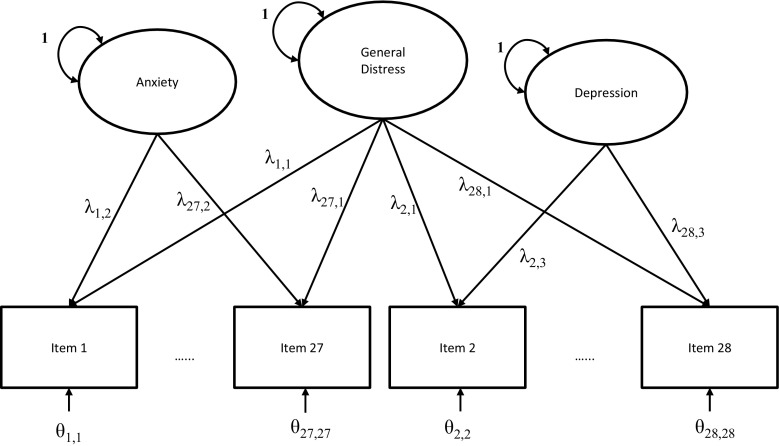
The bi-factor model on the HADS-items

All of the models were fitted using the metaSEM, and OpenMx packages in the R statistical platform. First we fit the Stage 1 and Stage 2 models with a fixed-effects model to the total set of studies (illustrating Case 1 from Table 1). The stage 1 analysis using the fixed-effects model involved fitting a model to the 28 correlation matrices in which all correlation coefficients were restricted to be equal across studies. Misfit of this model would indicate inequality of the correlation coefficients across studies. Stage 2 involved fitting the bi-factor model that Norton et al., (2013) found to have the best fit to the data (see Fig. 1).

Next, two subgroups of studies were created, one group with the 10 non-patient samples and the other with the 18 patient samples (illustrating Case 3 from Table 1). First, the Stage 1 analyses were performed in the two groups separately, leading to two pooled correlation matrices. Then, the factor model without equality constraints across subgroups was fitted to the data. Next, three models in which the factor loadings of the general distress factor, anxiety factor and depression factor respectively were constrained to be equal across patient and non-patient samples were tested. If the equality constraints on the factor loadings led to a significantly higher chi-square statistic, the (standardized) factor loadings would be considered to differ across groups.

Exact fit of a proposed model is rejected if the $\chi ^{2}$ statistic is found to be significant. Exact fit will rarely hold in MASEM, due to the large total sample size. Therefore, as in standard SEM, it is common to use approximate fit to assess the fit of models. Approximate close fit is associated with RMSEA-values under .05, satisfactory approximate fit with RMSEA-values under .08, and bad approximate fit is associated with RMSEA-values larger than .10 (MacCallum, Browne, & Sugawara, 1996). In addition to the RMSEA, we will evaluate the CFI (Bentler, 1990) and the standardized root mean squared residual (SRMR). CFI-values above .95 and SRMR-values under .08 are considered satisfactory (Hu & Bentler, 1999). For more information about the calculation and use of fit-indices in SEM we refer to Schermelleh-Engel et al., (2003).

#### Results

##### **Overall Stage 1: Testing homogeneity and pooling correlation matrices**

The Stage 1 model did not have exact fit to the data, $\chi ^{2}$ (2,457) = 10,400.04, p <.01. Approximate fit was acceptable according to the RMSEA (.064, 95% CI: [.063 ; .066]), but not according to the CFI (.914) and SRMR (.098). Based on the CFI and SRMR, one should not continue to fit the structural model, or use random-effects modeling. However, in order to illustrate the modeling involved in Case 1, we will continue with Stage 2 using overall fixed-effects analysis. Table 2 shows the pooled correlation matrix based on the fixed-effects Stage 1 analysis.

**Table 2:** Pooled correlation matrix based on the fixed effects Stage 1 analysis of the HADS data

	v1	v3	v5	v7	v9	v11	v13	v2	v4	v6	v8	v10	v12	v14
v1	1													
v3	.48	1												
v5	.55	.52	1											
v7	.42	.36	.41	1										
v9	.42	.46	.42	.35	1									
v11	.33	.29	.33	.32	.28	1								
v13	.49	.54	.50	.36	.50	.37	1							
v2	.29	.24	.30	.34	.25	.18	.26	1						
v4	.29	.28	.32	.36	.27	.18	.28	.42	1					
v6	.40	.36	.43	.40	.31	.22	.36	.38	.45	1				
v8	.35	.30	.34	.28	.27	.23	.33	.36	.25	.33	1			
v10	.23	.21	.25	.22	.18	.17	.22	.25	.26	.30	.26	1		
v12	.30	.27	.32	.36	.28	.19	.29	.47	.46	.42	.32	.33	1	
v14	.24	.22	.25	.34	.22	.21	.25	.28	.31	.31	.19	.21	.33	1

##### **Overall Stage 2: Fitting a factor model to the pooled correlation matrix**

Norton et al., (2013) concluded that a bi-factor model showed the best fit to the data. We replicated the analyses and found that, indeed, the model fit is acceptable according to the RMSEA (*χ*^2^(63) = 2,101.48, RMSEA = .039, 95% CI RMSEA: [.037 ; .040], CFI = .953, SRMR = .033). The parameter estimates from this model can be found in Table 3. All items loaded substantially on the general factor, and most items had smaller loadings on the specific factor. Contrary to expectations, Item 7 has a negative loading on the anxiety factor.

**Table 3:** Parameter estimates and 95% confidence intervals from the bi-factor model on the total HADS data

	**Λ** General			**Λ** Anxiety			**Λ** Depression			**Θ**		
	est.	lb	ub	est.	lb	ub	est.	lb	ub	est.	lb	ub
v1	.69	.68	.70	.19	.17	.22				.48	.47	.50
v3	.61	.60	.62	.40	.38	.42				.47	.45	.48
v5	.71	.70	.72	.23	.21	.26				.45	.44	.46
v7	.71	.70	.72	−.13	−.16	−.09				.48	.45	.50
v9	.56	.54	.57	.33	.31	.36				.58	.57	.59
v11	.48	.46	.49	.12	.10	.15				.76	.75	.77
v13	.63	.62	.64	.45	.42	.47				.40	.39	.42
v2	.47	.46	.48				.47	.45	.48	.56	.55	.57
v4	.50	.48	.51				.44	.42	.45	.56	.55	.58
v6	.61	.60	.63				.29	.28	.31	.54	.52	.55
v8	.50	.49	.52				.21	.19	.23	.70	.69	.71
v10	.37	.35	.38				.27	.25	.29	.79	.78	.80
v12	.50	.48	.51				.53	.51	.55	.47	.46	.49
v14	.43	.42	.44				.23	.21	.25	.76	.75	.77

Note: est = parameter estimate, lb = lower bound, ub = upper bound, **Λ** General, **Λ** Anxiety and **Λ** Depression refer to the factor loadings associated with these factors, **Θ** refers to residual variance

##### **Subgroup Stage 1: Testing homogeneity and pooling correlation matrices**

In the patient group, homogeneity was rejected by the chi-square test (*χ*^2^(1,547) = 5,756.84, p <.05). Homogeneity could be considered to hold approximately, based on the RMSEA (.071, 95% CI: [.070 ; .073]), but not based on the CFI (.923) and SRMR (.111). In the non-patient group, homogeneity was also rejected by the chi-square test, $\chi ^{2}$(819) = 3,254.60, p <.05, but approximate fit could be considered acceptable based on the RMSEA and SRMR (RMSEA = .049, 95% CI RMSEA: [.048 ; .051], CFI = .941, SRMR = .062). Although the model with a common correlation matrix does not have acceptable fit in the patient group, indicating that not all heterogeneity is explained by differentiating patient and non-patient samples, we continue with Stage 2 analysis as an illustration of the procedure when the interest is Case 2 (see Table 1).

##### **Subgroup Stage 2: Testing equality of factor loadings**

**Fig. 2:**
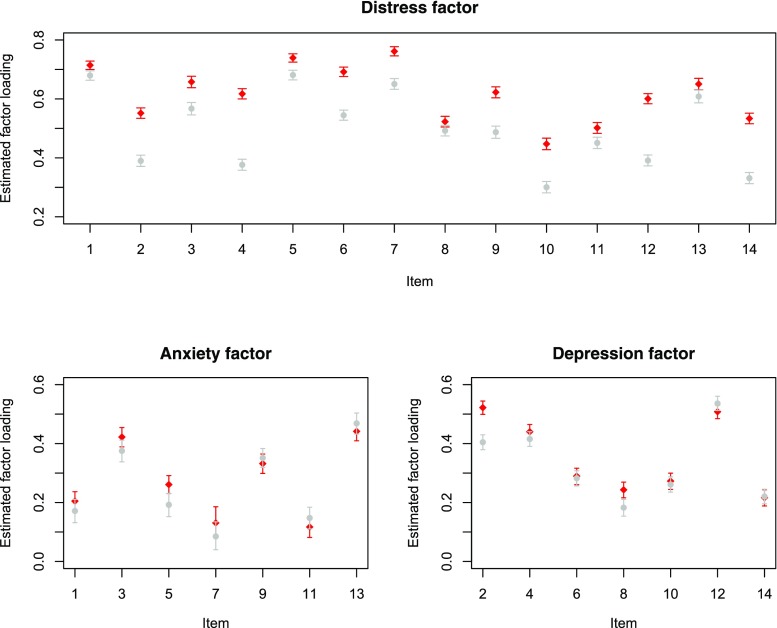
A plot of the estimated factor loadings and 95% confidence intervals for the patient group (red) and non-patient group (grey) Note: We show the absolute value of the factor loading of Item 7 on the Anxiety factor

The fit of the models with freely estimated factor loadings and with equality constraints on particular sets of factor loadings can be found in Table 4. The RMSEAs of all models indicated close approximate fit. However, the $\chi ^{2}$-difference tests show that the factor loadings cannot be considered equal for any of the three factors. Figure 2 shows a plot of the standardized factor loadings in the two groups. For the majority of the items, the factor loadings are higher in the patient group than in the non-patient group.

**Table 4:** Overall fit and difference in fit of the factor model with different equality constraints across groups

	df	$\chi ^2$	p	RMSEA [95% CI]	CFI	SRMR	Δdf	Δ*χ*^2^	p
1. No constraints	126	2249.21	<.05	.039 [.038 ; .041]	.955	.035			
2. **Λ** General equal	140	3125.51	<.05	.044 [.043 ; .046]	.936	.061	14	876.30	<.05
3. **Λ** Anxiety equal	133	2266.14	<.05	.038 [.037 ; .040]	.955	.036	7	16.93	<.05
4. **Λ** Depression equal	133	2300.62	<.05	.039 [.037 ; .040]	.954	.037	7	51.41	<.05

Note: Δdf and ${\Delta }\chi ^2$ refer to the difference in df and $\chi ^2$ in comparison with Model 1

#### Discussion

We found that the factor loadings of the bi-factor model on the HADS differed across the studies involving patients versus studies involving non-patients. The items were generally found to be more indicative of general distress in the studies with patient samples than in the studies with non-patient samples. A possible reason for this finding is that the HADS was developed for use in hospital settings, and thus was designed for use with patients. In practice, researchers may continue with the analysis by testing the equality of individual factor loadings across subgroups. For example, the factor loading of Item 2 from the Depression factor seems to differ more across groups than the other factor loadings for this factor. Such follow-up analyses may give more insight into specific differences across subgroups. However, it is advisable to apply some correction on the significance level, such as a Bonferroni correction, when testing the equality of several parameters individually.

A problem with these data is that the HADS is scored on a 4-point scale, but the analysis was performed on Pearson product moment correlations, assuming continuous variables. This may have led to underestimated correlation coefficients. Moreover, it would have been informative to analyze covariance matrices rather than correlation matrices, enabling a test on weak factorial invariance. However, the standard deviations were not available for most of the included studies.

We used fixed-effects overall and subgroup analysis, although homogeneity of correlation matrices did not hold. Therefore, it would have been more appropriate to apply random-effects analysis. However, due to the relatively large number of variables and the small number of studies, a random-effects model did not converge to a solution. Even the most restrictive model with only a diagonal $\mathbf {T}^{2}$ that was set to be equal across subgroups did not solve this problem. The results that were obtained should thus be interpreted with caution, as the Type 1 errors may be inflated. The next example shows random-effects subgroup-analysis, which may be the appropriate framework in most cases.

### Example 2 – Testing moderation of the effect of teacher-student relations on engagement and achievement

#### Introduction

In this example we use random-effects subgroup analysis to test moderation by SES in a path model linking teacher-child relations to engagement and achievement. Children with low SES are often found to be at risk of failing in school and dropping out (Becker and Luthar, 2002). According to Hamre and Pianta (2001), children at risk of failing in school may have more to gain from an ability to adapt to the social environment of the classroom than children who are doing very well at school. Therefore, it can be expected that the effects of teacher-child relations may be stronger for children with lower SES.

Roorda, Koomen, Spilt, and Oort (2011) performed a meta-analysis on correlation coefficients between measures of positive and negative teacher-student relations, engagement and achievement. They used univariate moderator analysis, and found that all correlations were larger in absolute value for studies with relatively more students with low SES. In the current analysis, we will test the moderation of the specific effects in a path model. We will use 45 studies reported by Roorda et al., (2011) and Jak, Oort, Roorda, and Koomen (2013), which include information about SES of the samples.

#### Analysis

**Fig. 3:**
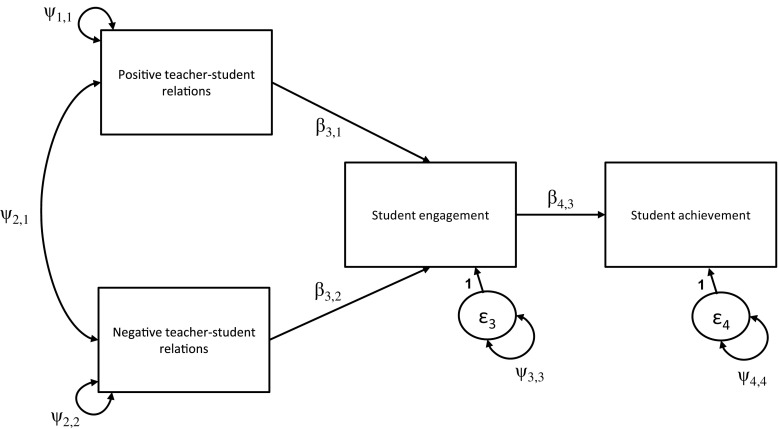
The hypothesized path model for Example 2

First we will perform a random-effects Stage 1 and Stage 2 analysis on the total sample of studies (representing Case 2 from Table 1). Next, we split the studies into two subgroups based on SES (representing Case 4 from Table 1). We will fit the hypothesized path model (see Fig. 3) to a group of studies in which the majority of the respondents were indicated to have low SES (24 studies), and a group of studies for which the majority of the sample was indicated with high SES (21 studies). Note that SES is a continuous moderator variable in this case (percentages). We split the studies in two groups based on the criterion of 50% of the sample having low SES. Then, we test the equivalence of the direct effects across groups by constraining the effects to be equal across subgroups. Using a significance level of .05, if the $\chi ^{2}$ statistic increased significantly given the increased degrees of freedom when adding equality constraints across groups, one or more of the parameters would be considered significantly different across groups. Note that dichotomizing a continuous variable is generally not advised. In this example we dichotomize the moderator in order to illustrate subgroup analyses. Moreover, in TSSEM, the analysis of continuous moderator variables is not yet well developed.

#### Results

##### **Overall Stage 1: Random-effects analysis**

The pooled correlations based on the random-effects analysis can be found in Table 5. When a random-effects model is used, an $I^{2}$ value may be calculated. It can be interpreted as the proportion of study-level variance in the effect size (Higgins & Thompson, 2002). The $I^{2}$ values (above the diagonal) show that there is substantial between-studies variability in the correlation coefficients, ranging from .79 to .94.

**Table 5:** Pooled correlations (under the diagonal) and $I^2$ (above the diagonal) based on the random effects Stage 1 analysis

	v1	v2	v3	v4
v1. Positive relations	1	.92	.94	.79
v2. Negative relations	−.24	1	.88	.80
v3. Engagement	.32	−.31	1	.90
v4. Achievement	.14	−.18	.28	1

##### **Overall Stage 2: Fitting a path model**

We fitted a path model to the pooled Stage 1 correlation matrix, in which positive and negative relations predicted achievement indirectly, through engagement. Exact fit of this model was rejected (*χ*^2^ (2) = 11.16, p <.05). However, the RMSEA of .013 (95% CI = [.006 ; .020]) indicated close approximate fit, as well as the CFI (.966) and SRMR (.045). Table 6 shows the parameter estimates and the associated 95% confidence intervals. All parameter estimates were considered significantly different from zero, as zero is not included in the 95% confidence intervals. The indirect effects of positive and negative relations on achievement were small, but significant. Although the model shows good fit on the averaged correlation matrix, this analysis provides no information about whether SES might explain the between-study heterogeneity. Subgroup analysis is used to test whether the parameters differ across studies with different levels of average SES.

**Table 6:** Parameter estimates and 95% confidence intervals of the hypothesized path model

Parameter	est	lb	ub
$\beta _{31}$	.27	.20	.35
$\beta _{32}$	−.30	−.38	−.22
$\beta _{43}$	.35	.29	.41
$\beta _{31}$ * *β*_43_	.10	.07	.12
$\beta _{32}$ * $\beta _{43}$	−.10	−.14	−.07
$\psi _{12}$	−.24	−.32	−.16
$\psi _{33}$	.80	.73	.85
$\psi _{44}$	.88	.83	.92

##### **Subgroup Stage 1: Random-effects analysis**

Different pooled correlation matrices were estimated in the group of studies with low SES and the group of studies with high SES (see Tables 7 and 8). The proportions of between-studies variance (*I*^2^) within the subgroups are smaller than they were in the total sample, indicating that SES explains part of the between-study heterogeneity.

**Table 7:** Pooled correlations (under the diagonal) and $I^2$ (above the diagonal) based on the random effects Stage 1 analysis in studies with low SES

	v1	v2	v3	v4
v1. Positive relations	1	.85	.94	.71
v2. Negative relations	−.33	1	.83	.73
v3. Engagement	.35	−.35	1	.86
v4. Achievement	.12	−.18	.23	1

**Table 8:** Pooled correlations (under the diagonal) and $I^2$ (above the diagonal) based on the random effects Stage 1 analysis in studies with high SES

	v1	v2	v3	v4
v1. Positive relations	1	.90	.84	.79
v2. Negative relations	−.17	1	.66	.80
v3. Engagement	.23	−.23	1	.87
v4. Achievement	.16	−.18	.34	1

##### **Subgroup Stage 2: Testing moderation of effects by SES**

The hypothesized path model showed acceptable approximate fit, but no exact fit, in the low-SES group, $\chi ^{2}$ (2) = 6.28, p <.05, RMSEA = .013 (95% CI = [.002 ; .026]), CFI = .978, SRMR = .041 as well as in the high-SES group, $\chi ^{2}$ (2) = 9.50, p <.05, RMSEA = .015 (95% CI = [.006 ; .025]), CFI = .936, SRMR = .0549. The fit of the unconstrained baseline model, with which the fit of the models with equality constraints will be compared, is equal to the sum of the fit of the models in the two subgroups. Therefore, the $\chi ^{2}$ and df against which the constrained models will be tested is df = 2 + 2 = 4 and $\chi ^{2}$ = 6.28 + 9.50 = 15.78. Constraining the three direct effects in the path model to be equal across subgroups did not lead to a significant increase in misfit, ${\Delta }\chi ^{2}$ (3) = 5.18, p = .16. Therefore, the null hypothesis of equal direct effects across subgroups is not rejected.

##### **Discussion**

In this example we tested whether the direct effects in a path model linking teacher-child relations to engagement and achievement were moderated by SES. The subgroup analysis showed that the null-hypothesis stating that the effects are equal in the low SES and high SES populations cannot be rejected. Note that non-rejection of a null-hypothesis does not imply that the null-hypothesis is true. It could also mean that our design did not have enough statistical power to detect an existing difference in the population.

## Simulation study

It is often necessary to create subgroups of studies, because an overall analysis will mask differences in parameters across subgroups. For example, if the population regression coefficient is 0.20 for Subgroup 1, and 0.30 for Subgroup 2, an analysis of all of the studies together will result in an estimated regression coefficient of between 0.20 and 0.30. This means that the effect will be overestimated for Subgroup 1 and underestimated for Subgroup 2. Subgroup analysis will lead to better parameter estimates in the subgroups. However, creating subgroups may lead to small numbers of studies within each subgroup. In combination with having twice as many parameters to be estimated as with an overall analysis, small numbers of studies will likely result in estimation problems such as non-convergence. Convergence is an important issue, because researchers will be unable to present any meaningful results of the MASEM analysis without having a converged solution. In order to evaluate the effect of the number of studies within each subgroup on the frequency of estimation problems, we conducted a small simulation study.

### Data generation and conditions

We generated data from two subgroups, in which one regression coefficient differed by .10 points across subgroups in the population. Next, we fitted the correct model to the two subgroups separately, as well as to the combined data. We expected that, due to the larger number of studies, the percentage of converged solutions would be larger for the overall analysis than for the subgroup analyses and that the estimation bias in the manipulated effect would be smaller in the subgroup analysis (because the regression coefficient is allowed to be different in each subgroup).

The data-generating model was based on the results from Example 2. The population values for the direct effects in Subgroup 1 were: $\beta _{31}$ = .265, $\beta _{32}$ = -.307, $\beta _{43}$ = .288, and $\psi _{31}$ = -.329. The between-studies variance used to generate random correlation matrices was based on Example 2. In Subgroup 2, all population values were identical to the values in Subgroup 1, except for $\beta _{43}$, which was .388 (.10 larger than in Subgroup 1). We generated data with $k = 22$, $k = 44$, $k = 66$ or $k = 88$ studies per subgroup, with sample sizes of n = 200 for each study. For each condition we generated 2000 meta-analytic datasets.

In each condition we fitted the correct model to the two subgroups separately, as well as to the subgroups combined. We restricted the between-studies covariance matrices to be diagonal, in order to reduce the number of parameters to be estimated. In practice, this restriction is often applied (Becker, 2009). We evaluated the percentage of converged solutions, the relative bias in the estimate of $\beta _{43}$, and the relative bias in the standard error of $\beta _{43}$ across methods and conditions. The relative percentage of estimation bias for $\beta _{43}$ was calculated as

4 $$ 100 * \frac{\hat{\beta}_{43} - \beta_{43}} {\beta_{43}}. $$

We regarded estimation bias of less than 5% as acceptable (Hoogland & Boomsma, 1998). The relative percentage of bias in the standard error of $\beta _{43}$ was calculated as:

5 $$ 100 * \frac{\bar{SE}(\hat{\beta}_{43}) - SD(\hat{\beta}_{43})} {SD(\hat{\beta}_{43})}, $$

where $\bar {SE}(\hat {\beta }_{43})$ is the average standard error of $\hat {\beta }_{43}$ across replications, and $SD(\hat {\beta _{43}})$ is the standard deviation of the parameter estimates across replications. We considered the standard errors to be unbiased if the relative bias was smaller than 10% (Hoogland & Boomsma, 1998).

### Results

#### Convergence

**Fig. 4:**
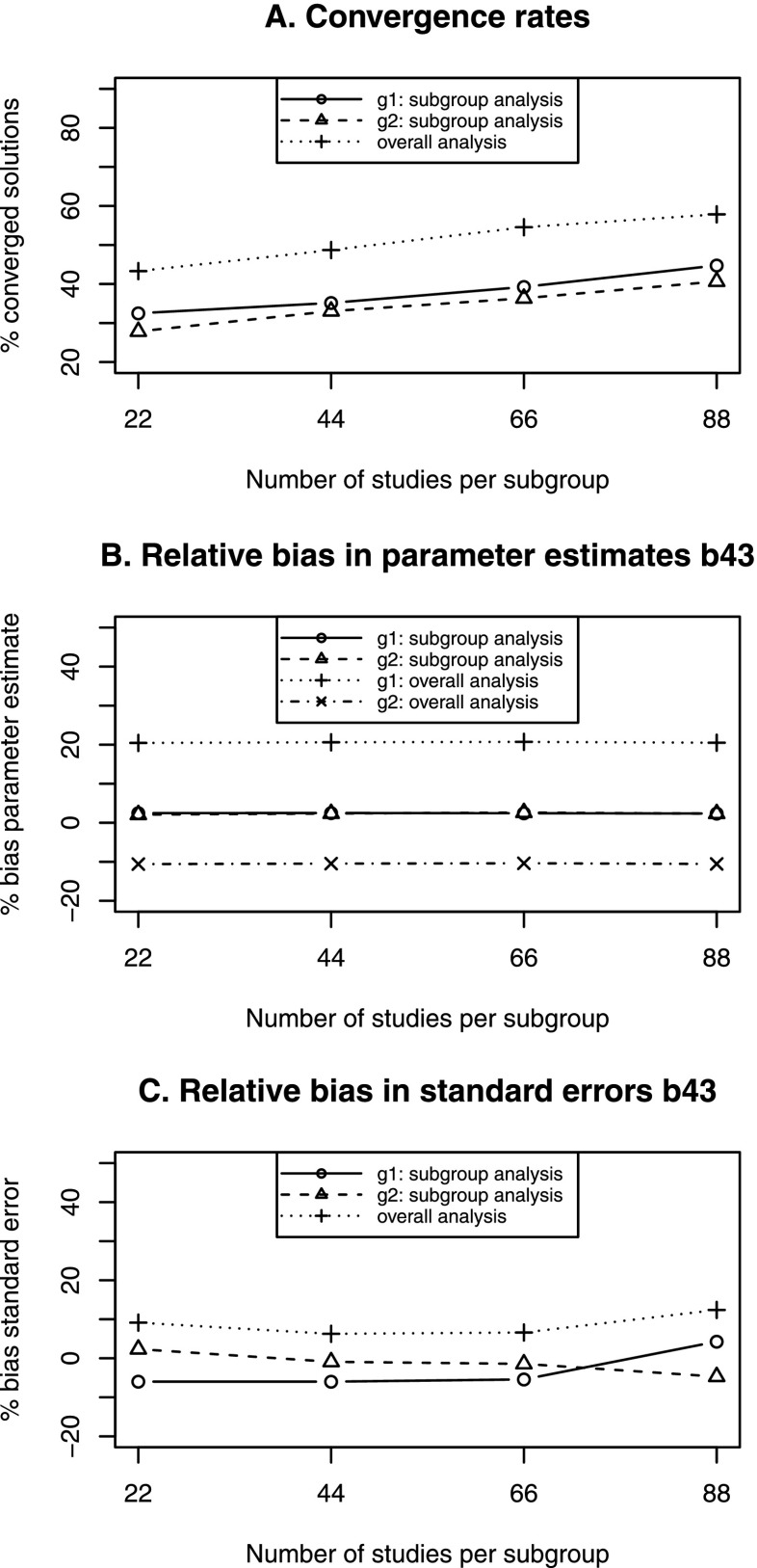
Convergence, parameter bias and standard error bias for overall and subgroup analysis with a group difference of 0.10 in *β*_43_ Note: The results in panels B and C are based on only those replications that led to a converged solution for all three analyses. The numbers of replications used are 141, 188, 246, and 300 replications for k = 22, k = 44, k = 66, and k = 88 respectively

Figure 4a shows the convergence rates for all conditions. As expected, the analysis of the total dataset resulted in more converged solutions than the subgroup analysis in all conditions. In addition, convergence rates increased with the number of studies. However, the convergence rates were generally low. For example, with 22 studies per subgroup (the condition similar to that of our Example 2), only 43% of the datasets led to a converged solution with the overall analysis, while only around 30% converged with the subgroup analysis. With small numbers of studies per subgroups (smaller than 44), most analyses are expected to not result in a converged solution.

#### Bias in parameter estimates

We evaluated the parameter bias in $\beta _{43}$ only for.^1^ The results are presented in Fig. 4b. The percentage of estimation bias was not related to the number of studies or to sample size. As expected, the overall analysis resulted in underestimation for Subgroup 1 and overestimation for Subgroup 2, while the subgroup analysis led to unbiased parameter estimates. Although the difference in the population value was only 0.10, the percentages of the relative bias exceeded the cut-off of 5% in all conditions for the overall analysis. For parameters that did not differ across subgroups, all analyses yielded unbiased estimates.

#### Bias in standard errors

The relative bias in standard errors was around 10% in all conditions for the overall analysis. With the subgroup analysis, the standard error estimates were more accurate, with a bias of between roughly -5% and 5% in all conditions. The results are presented in Fig. 4c. The standard errors of the parameters that did not differ across subgroups were unbiased for all analyses.

### Conclusion on the simulation study

The simulation study showed that convergence is a serious potential problem when applying random-effects MASEM. Moreover, the likelihood of non-convergence occurring increases with smaller numbers of studies, such as with a subgroup analysis. However, *if* the model converges, the subgroup analysis will lead to better parameter estimates and standard error estimates in cases where a difference in the population coefficient is present, even if the population difference is small. In order to increase the likelihood of obtaining a converged solution, it is recommended that as many studies as possible be included.

## General discussion

We proposed subgroup analysis to test moderation hypotheses on specific parameters in MASEM. We illustrated the approach using TSSEM. The subgroup analysis method that was presented is not restricted to TSSEM. One could just as easily apply the subgroups analysis on pooled correlation matrices obtained with univariate approaches (Hunter & Schmidt, 2015; Hedges & Olkin, 1985) or the multivariate GLS-approach (Becker, 1992; 1995). However, based on earlier research comparing these approaches (Cheung & Chan, 2005b; Jak & Cheung, 2017), univariate approaches are not recommended for MASEM.

Creating subgroups of studies to test the equality of parameters across groups is a useful approach, but may also lead to relatively small numbers of studies within each subgroup. Given the large number of parameters involved in random-effects modeling, the number of studies may become too small for a converged solution to be obtained, as was the case in our Example 1. One way to reduce the number of parameters is to estimate the between-study heterogeneity variances but not the covariances among the random effects, i.e., restricting $\mathbf {T}^{2}$ to be diagonal. In practice, this restriction is often needed (Becker, 2009). We applied this constraint to the two subgroups in the second example and in the simulation study.

In the simulation study, we found that even with a diagonal heterogeneity matrix, random-effects subgroup modeling is often not feasible due to convergence problems. In practice, researchers may therefore have no other option than to apply fixed-effects modeling instead of random-effects modeling. However, ignoring between-study heterogeneity is known to lead to inflated false positive rates for significance tests (Hafdahl, 2008; Zhang, 2011). Researchers should therefore be careful when interpreting the results of significance tests in cases where heterogeneity exists but a fixed-effects model is applied. Collecting more studies to be included in the meta-analysis is preferable over switching to a fixed-effects model.

A limitation of the subgroup analysis to test moderation is that the moderator variables have to be categorical. In the second example, we split the studies into two groups based on the percentage of respondents with high SES in the study. By dichotomizing this variable we throw away information and lose statistical power. Indeed, contrary to our findings, the univariate meta-regression analyses reported by Roorda et al. showed significant moderation by SES. However, these analyses did not take into account the multivariate nature of the data, and tested the moderation of the correlation coefficients and not of the regression coefficients. Future research is needed to develop methods to include study-level variables as continuous covariates in TSSEM.

### Concluding remarks

In the current paper we presented a framework to test hypotheses about subgroup differences in meta-analytic structural equation modeling. The metaSEM and OpenMx-code and R-functions used in the illustrations are provided online, so that researchers may easily adopt the proposed procedures to test moderator hypotheses in their MASEM analyses. The simulation study showed that increasing the number of studies in a random-effects subgroup analysis increases the likelihood of obtaining a converged solution.

## Footnotes

1 Consequently, the numbers of replications used to calculate the bias were 141, 188, 246, and 300 of the 2000 replications for k = 22, k = 44, k = 66, and k = 88, respectively. We have also calculated the bias using all converged solutions per method (resulting in larger, but different numbers of replications being used for different analyses). This approach leads to very similar results, and identical conclusions.

## References

[CR1] Becker BE, Luthar SS (2002). Social-emotional factors affecting achievement outcomes among disadvantaged students: Closing the achievement gap. Educational Psychologist.

[CR2] Becker B (1992). Using results from replicated studies to estimate linear models. Journal of Educational Statistics.

[CR3] Becker B (1995). Corrections to using results from replicated studies to estimate linear models. Journal of Educational and Behavioral Statistics.

[CR4] Becker, B. J. (2009). Model-based meta-analysis. In Cooper, H., Hedges, L. V., & J C Valentine (Eds.) *The handbook of research synthesis and meta-analysis*. (2nd edn.) (pp. 377–395). New York: Russell Sage Foundation.

[CR5] Bentler P (1990). Comparative fit indexes in structural models. Psychological Bulletin.

[CR6] Bentler P (2007). Can scientifically useful hypotheses be tested with correlations?. The American Psychologist.

[CR7] Bentler, P. M., & Savalei, V. (2010). Analysis of correlation structures: Current status and open problems. In Kolenikov, S., Steinley, D., & Thombs L. (Eds.) *Statistics in the Social Sciences* (pp. 1–36). New Jersey: Wiley.

[CR8] Boker, S. M., Neale, M. C., Maes, H. H., Wilde, M. J., Spiegel, M., Brick, T. R., & BDBL OpenMx, T. (2014). Openmx 2.0 user guide [Computer software manual].

[CR9] Borenstein M, Hedges LV, Higgins JPT, Rothstein H (2009). Introduction to meta-analysis.

[CR10] Browne M (1984). Asymptotically distribution-free methods for the analysis of covariance structures. British Journal of Mathematical and Statistical Psychology.

[CR11] Cheung, M. W.-L., & Chan, W. (2005a). Classifying correlation matrices into relatively homogeneous subgroups: a cluster analytic approach. *Educational and Psychological Measurement*, *65* (6), 954–979. 10.1177/0013164404273946

[CR12] Cheung MW-L, Chan W (2005). Meta-analytic structural equation modeling: A two-stage approach. Psychological Methods.

[CR13] Cheung M (2009). Comparison of methods for constructing confidence intervals of standardized indirect effects. Behavior Research Methods.

[CR14] Cheung MW-L, Chan W (2009). A two-stage approach to synthesizing covariance matrices in meta-analytic structural equation modeling. Structural Equation Modeling: A Multidisciplinary Journal.

[CR15] Cheung M (2014). Fixed- and random-effects meta-analytic structural equation modeling: Examples and analyses inR. Behavior Research Methods.

[CR16] Cheung M (2015). Meta-analysis: A structural equation modeling approach.

[CR17] Cheung, M. W. -L. (2015). metaSEM: An R package for meta-analysis using structural equation modeling. Frontiers in Psychology, 5(1521). 10.3389/fpsyg.2014.01521 10.3389/fpsyg.2014.01521 PMC4283449 25601849

[CR18] Cheung MW-L, Cheung S (2016). Random-effects models for meta-analytic structural equation modeling: Review, issues, and illustrations. Research synthesis methods.

[CR19] Drees JM, Heugens PPMA (2013). Synthesizing and extending resource dependence theory a meta-analysis. Journal of Management.

[CR20] Earnest DR, Allen DG, Landis R (2011). Mechanisms linking realistic job previews with turnover: A meta-analytic path analysis. Personnel Psychology.

[CR21] Gerow JE, Ayyagari R, Thatcher JB, Roth PL (2013). Can we have fun @ work? the role of intrinsic motivation for utilitarian systems. European Journal of Information Systems.

[CR22] Hafdahl A (2008). Combining heterogeneous correlation matrices: Simulation analysis of fixed-effects methods. Journal of Educational and Behavioral Statistics.

[CR23] Hamre BK, Pianta R (2001). Early teacher–child relationships and the trajectory of children’s school outcomes through eighth grade. Child Development.

[CR24] Haus I, Steinmetz H, Isidor R, Kabst R (2013). Gender effects on entrepreneurial intention: A meta-analytical structural equation model. International Journal of Gender and Entrepreneurship.

[CR25] Hedges L, Olkin I (1985). Statistical methods for meta-analysis.

[CR26] Hedges L, Vevea J (1998). Fixed- and random-effects models in meta-analysis. Psychological Methods.

[CR27] Higgins JPT, Thompson S (2002). Quantifying heterogeneity in a meta-analysis. Statistics in Medicine.

[CR28] Hoogland JJ, Boomsma A (1998). Robustness studies in covariance structure modeling an overview and a meta-analysis. Sociological Methods & Research.

[CR29] Hu L-t, Bentler P (1999). Cutoff criteria for fit indexes in covariance structure analysis: Conventional criteria versus new alternatives. Structural Equation Modeling.

[CR30] Hunter JE, Hamilton M (2002). The advantages of using standardized scores in causal analysis. Human Communication Research.

[CR31] Hunter JE, Schmidt F (2015). Methods of meta-analysis: correcting error and bias in research findings.

[CR32] Jak S, Oort FJ, Roorda DL, Koomen H (2013). Meta-analytic structural equation modelling with missing correlations. Netherlands Journal of Psychology.

[CR33] Jak S (2015). Meta-analytic structural equation modeling.

[CR34] Jak, S., & Cheung, M. W. -L. (2017). Accounting for missing correlation coefficients in fixed-effects meta-analytic structural equation modeling. Multivariate Behavioral Research, in press. 10.1080/00273171.2017.1375886 29220593

[CR35] Jiang K, Liu D, Mckay PF, Lee TW, Mitchell TR (2012). When and how is job embeddedness predictive of turnover? A meta-analytic investigation. Journal of Applied Psychology.

[CR36] Kwan JLY, Chan W (2011). Comparing standardized coefficients in structural equation modeling: a model reparameterization approach. Behavior Research Methods.

[CR37] Lipsey M, Wilson D (2001). Practical meta-analysis.

[CR38] MacCallum RC, Browne MW, Sugawara HM (1996). Power analysis and determination of sample size for covariance structure modeling. Psychological Methods.

[CR39] Meredith W (1993). Measurement invariance, factor analysis and factorial invariance. Psychometrika.

[CR40] Mitchell AJ, Meader N, Symonds P (2010). Diagnostic validity of the hospital anxiety and depression scale (HADS) in cancer and palliative settings: A meta-analysis. Journal of Affective Disorders.

[CR41] Norton S, Cosco T, Doyle F, Done J, Sacker A (2013). The hospital anxiety and depression scale: A meta confirmatory factor analysis. Journal of Psychosomatic Research.

[CR42] R Core Team (2017). R: A language and environment for statistical computing. R Foundation for Statistical Computing. Retrieved from http://www.R-project.org

[CR43] Roorda DL, Koomen HMY, Spilt JL, Oort F (2011). The influence of affective teacher-student relationships on students’ school engagement and achievement: a meta-analytic approach. Review of Educational Research.

[CR44] Rosenbusch N, Rauch A, Bausch A (2013). The mediating role of entrepreneurial orientation in the task environment–performance relationship a meta-analysis. Journal of Management.

[CR45] Schermelleh-Engel K, Moosbrugger H, Müller H (2003). Evaluating the fit of structural equation models: Tests of significance and descriptive goodness-of-fit measures. Methods of psychological research online.

[CR46] Steiger J (2002). When constraints interact: A caution about reference variables, identification constraints and scale dependencies in structural equation modeling. Psychological methods.

[CR47] van den Boer M, van Bergen E, de Jong P (2014). Underlying skills of oral and silent reading. Journal of experimental child psychology.

[CR48] Viswesvaran C, Ones D (1995). Theory testing: Combining psychometric meta-analysis and structural equations modeling. Personnel Psychology.

[CR49] Zakrzewska J (2012). Should we still use the hospital anxiety and depression scale?. Pain.

[CR50] Zhang Y (2011). Meta-analytic Structural Equation Modeling (MASEM): Comparison of the multivariate methods (phdthesis).

[CR51] Zigmond AS, Snaith R (1983). The hospital anxiety and depression scale. Acta Psychiatrica Scandinavica.

